# Stand-on ride-on power mobility devices for children with cerebral palsy: pilot study protocol for waitlist-control pre-post biomechanical changes

**DOI:** 10.3389/fped.2026.1761608

**Published:** 2026-03-12

**Authors:** Guilherme Manna Cesar, Ligia Yumi Mochida, Kira Flanagan, Kevin McDonald, Jasper Xu, Debra Depto-Hoffman, Juan Aceros

**Affiliations:** 1 Department of Physical Therapy, University of North Florida, Jacksonville, FL, United States; 2 Department of Health Administration, University of North Florida, Jacksonville, FL, United States; 3 Department of Electrical Engineering, University of North Florida, Jacksonville, FL, United States

**Keywords:** balance, cerebral palsy, clinical trial, early intervention, mobility, pediatric disability, power mobility device, waitlist control

## Abstract

**Background:**

Many children with cerebral palsy are often deprived of participation in life activities due to limited postural control and walking capabilities. Ride-on power mobility devices (PMD) allow for the self-generated, active control of mobility. A novel stand-on ride-on device may provide the additional support to maintaining upright posture to improve children's balance and lower extremity strength. This pilot clinical trial aims primarily to assess the feasibility of conducting a full-scale randomized controlled trial, including evaluation of recruitment, retention, adherence to the intervention, and variability in outcome measures. Secondarily, the study will explore biomechanical factors underlying potential changes in balance and mobility function following adapted stand-on PMD use in natural environments.

**Methods:**

Ten children will participate in this single-arm waitlist-controlled pre-post feasibility trial. Children 4–6 years old with spastic diplegia cerebral palsy (Gross Motor Function Classification System level III) will engage in the intervention with individually adapted stand-on PMD use for three months at home, neighborhood, and family places. Biomechanical assessment will take place at baseline (3 months prior to intervention), at pre-, and at post-intervention. Exploratory outcome measures include center of pressure variables, kinematic variables of full-body coordination, and lower extremity muscle co-contraction during gait, sit-to-stand, and balance tasks.

**Discussion:**

This pilot clinical trial is the first to evaluate biomechanical factors underlying changes in balance (static and dynamic) and mobility function after stand-on PMD intervention. By collecting key feasibility metrics and quantifying variability in biomechanical outcomes, this study will generate fundamental knowledge to guide the design of a larger, adequately powered trial that can validate PMD interventions in decreasing the lifelong burden of secondary medical conditions that emerge in this population due to impaired balance and limited mobility. Findings will also provide the empirical data supporting the follow-up grant application with larger-scale study design.

**Clinical Trial Registration:**

ClinicalTrials.gov, identifier NCT06455930.

## Introduction

Children's participation in life activities with family and peers plays a vital role in their physical and psychosocial development; however, many children with neurologic-induced physical disabilities are often deprived of such interactions given limited postural control (1, 2) and walking capabilities, leading to delayed development and reduced quality of life (3, 4). In the past ∼10 years, ride-on power mobility devices (PMD), such as the go-baby-go cars (5–8), have gained much public health attention. These devices allow for the self-generated, active control of mobility, spatial exploration, and socialization that many of these children need (9).

Significant improvements in qualitative measures involving several areas of development have been reported for children with impaired mobility from varying developmental disabilities when PMD interventions were provided (7, 10–14). In particular, work stemming from the Go-Baby-Go initiative has consistently demonstrated meaningful improvements in psychosocial outcomes, including participation, social engagement, and quality of life, in children receiving individually adapted ride-on mobility devices (6, 7, 14, 15). While seated ride-on PMDs provide the self-directed exploration in alignment with the child's optic flow, they do not provide the link between the novel motor behavior and sensory experience, i.e., the child is moving but not activating lower extremity muscles and receiving information from mechanoreceptors to maintain standing posture and manipulate balance demands. More recently, adapted stand-on ride-on PMDs (10, 16, 17) have provided the additional support to maintaining an upright posture, increasing lower-extremity loading and proprioceptive demands that are absent during seated mobility. Prior work indicates that task-specific standing interventions can improve both static and dynamic balance in children with cerebral palsy, as evidenced by reductions in center-of-pressure sway following three-month weight-shifting programs (18, 19). In parallel, functional standing activities have been shown to enhance lower-extremity muscle strength and endurance, which are critical contributors to postural control during gait (20, 21). Despite these promising findings, the biomechanical mechanisms underlying balance and mobility changes associated with stand-on power mobility interventions remain largely unexplored.

Besides the limited scope of case reports (6, 22, 23) and case series (8, 24, 25) for the seated PMD, literature has largely provided only qualitative outcomes after use of these devices. Although important to support feasibility of administering interventions with devices for young children with disabilities to support self-directed mobility and socialization (6, 7, 10, 15, 22), available data do not elucidate the effects of powered mobility intervention on sensory-motor impairments, such as postural stability and whole-body motor coordination during static and dynamic conditions. The biomechanical factors underlying the purported beneficial physical changes of using stand-on PMDs have not been explored and are yet unknown. Biomechanical analysis enables objective quantification of postural stability, whole-body coordination, and neuromuscular control by integrating kinetic, kinematic, and electromyographic measures, thereby enabling mechanistic characterization of how mobility-based interventions may influence balance and movement strategies (2). Understanding which biomechanical factors can be altered after PMD use, especially stand-on devices, will guide subsequent intervention work to enhance functional outcomes (26).

This manuscript describes a study protocol for a pilot feasibility clinical trial designed to generate needed preliminary data to support subsequent adequately powered randomized controlled trial (RCT). The study is not intended to test intervention efficacy, but rather it aims to characterize feasibility metrics and explore variability in biomechanical outcomes associated with adapted stand-on PMD use in children with cerebral palsy and limited walking and balance control. Lifelong disabilities resulting from cerebral palsy lead to increased healthcare costs and place additional strain on caregivers (27). Our long-term research goal is to develop and validate effective interventions that can enhance the quality of life for children with cerebral palsy and their families. This aligns with the missions of National Institutes of Health (NIH)'s Eunice Kennedy Shriver National Institute of Child Health and Human Development (NICHD) and National Center for Medical Rehabilitation Research (NCMRR) to advance fundamental knowledge about the nervous system and apply that knowledge to alleviate the burden of neurological diseases.

### Objectives

The objective is to describe the study protocol of a pilot feasibility clinical trial. In this foundational work, we will test the feasibility of administering to a cohort of children with cerebral palsy an intervention with individually adapted stand-on PMD for three months.

#### Primary objective

To determine the feasibility of conducting a future RCT evaluating adapted stand-on PMD in young children with cerebral palsy [Gross Motor Function Classification System (GMFCS) level III] by assessing recruitment rates, retention, adherence to intervention, and variability in key outcome measures.

#### Secondary (exploratory) objective

To descriptively characterize changes in biomechanical measures related to static balance, dynamic balance, and gait following three months of adapted stand-on PMD use, with the purpose of informing outcome selection and sample-size estimation for future trials.

## Methods

### Study design

This design of the presented study protocol adheres to the established definition of a pilot/feasibility study, as outlined by the CONSORT extension for pilot and feasibility studies. Additionally, given the pilot nature of this project (R03) and the population (4–6-year-old children) who can undergo spurs of physical maturation, we selected a waitlist control design in which a baseline evaluation is administered 3 months prior to the pre-intervention evaluation. This is performed to control for changes in the secondary outcome measures that are due to maturation and not the proposed intervention.

### Study setting

While assessments are performed in the biomechanics laboratory within the university, intervention will take place at the children's home, neighborhood, and familiar places where the individually adapted PMD can be used.

### Participants

Study inclusion criteria {10} include: 1) Diagnosis of spastic diplegia cerebral palsy, GMFCS level III (28); 2) between 4 and 6 years old (age band selected based on the benefits of early intervention (12, 24) and the age-limitation imposed from the size constraints of the PMDs (toy cars) selected for the intervention); 3) able to stand and to walk short distances (at least 10 meters) with or without external assistance; 4) physician medical clearance to participate; and 5) exhibit sufficient cooperation and attention so that simple verbal instructions can be followed. Potential participants will be excluded if they: 1) have notable orthopedic conditions (e.g., lower extremity amputation; recent hip surgeries or soft tissue lengthening); 2) inadequate vision to complete study's procedures safely; 3) serious/unstable cardiac conditions that prevent engagement in the Evaluation Sessions; and 4) any other factor that might hinder full participation in the study or confound interpretation of the results. Although we will aim to recruit children who are not receiving balance/walking-specific rehabilitation, children can be engaged in therapy sessions while taking part in the study. We will account for the type and amount of rehabilitation statistically to maintain rigor of research and to generate robust homogeneous findings to support the subsequent study.

Ten children will be recruited {15} from local rehabilitation programs. Although no sex-difference in balance performance has been reported for children with cerebral palsy at the age band of interest (29), smaller differences in trunk control have been reported for children with cerebral palsy ages 8–14 years (30) and typically developing children (31, 32). Thus, we will aim to recruit five boys and five girls since sex will be considered as a biological factor (33–35) in the statistical analyses of the subsequent larger grant application.

### Intake screening and consent/assent procedures

Once potential participants contact us, the initial intake screening to confirm eligibility will be performed by the research team's physical therapist who has 30 + years of experience with evaluating and treating children with neurologic condition, including cerebral palsy. This intake, which takes place at the Laboratory of Applied Biomechanics and Engineering at the University of North Florida, involves assessment of each participants’ anthropometric measurements, range of motion, and muscle tone. Weight (kg) will be measured using a calibrated digital scale (Pelstar Professional Scale, model 349KLX, China) with participants standing independently when possible, or seated when standing is not feasible. Height (cm) will be measured using a wall-mounted stadiometer (Seca, model 264, Germany) with clinician assisting child to maintain a stand-up posture when needed. Following, with the child lying supine on a floor mat, lower-extremity and bilateral foot length (cm) will be measured using a tape measure; width (mm) of bilateral knees, ankles, and forefeet will be measured using a digital caliper. Passive range of motion will be assessed using a standard goniometer to measure joint angles accurately. Muscle tone will be assessed using the modified Ashworth Scale, which provides a reliable grading of spasticity levels in each of the joints of the participants’ extremities.

Once confirmed eligibility, parents and children will meet with the PI of the study (GMC) at a dedicated private room for the informed consent/assent procedures approved by the University's Institutional Review Board (UNF IRB LEG-2147539). A basic set of medical information will be reviewed to confirm participant's eligibility for the study and physician clearance for participation in the activities associated with the research study will be obtained. If a participant withdraws, we will document the reason for withdrawal and recruit a new participant to replace the individual who withdrew.

### Building and safety-check of individualized PMD

During the intake screening, key information regarding the child's disabilities, needs, handedness, and interests (e.g., color, cartoon/movie character) will be collected. Engineering students from Mechanical, Electrical, and Manufacturing programs will be recruited to participate in the research activities. After CITI certification, these students will modify the PMD under the guidance of an engineering faculty expert (JA, KF). The modifications include customizing the seating and control interface to match the child's physical and cognitive abilities, harness support system (child with or without gastrostomy tube), integrating safety features such as automatic shut-off mechanisms (parental control), and enhancing the device's stability and maneuverability. The ride-on toy will also be modified with a “sit-to-stop” design, in which a power interrupter switch is placed in series with the device's power connection; when pressed down (i.e., when child sits), this switch located under the seat stops the ride-on from moving.

Prior to delivering the PMD to the child, a safety checklist including electrical and mechanical items will be employed. Such items include questions regarding battery modifications, proper wiring (gauge, connections, insulation), rating for switches and other electronic components, structural elements and modifications, harness and support systems, no loose components, no sharp objects, or burrs (cuts, drill holes), and ensuring that the system can operate continuously for 10 min with a 40-pound weight without overheating.

A dedicated time will be allotted for parents/child safety and harness donning training. This type of training will be comparable to previous employed training for the adaptive ride-on program at the University (36). It consists of hand-over-hand guidance to briefly demonstrate to the child the cause-and-effect relationship between the activation interface and toy movement. Simple one-word verbal labels are used in conjunction with the haptic guidance, e.g., Go, Stop, and Push. Following this, the child is given the opportunity for random, free exploration of the motor activation and the consequent movement of the mobility device without further adult instruction. In addition, instruction on how to take care of the device (cleaning, charging) will be presented to parents/guardians.

### Intervention protocol and tracking of PMD usage

**Figure 1:**
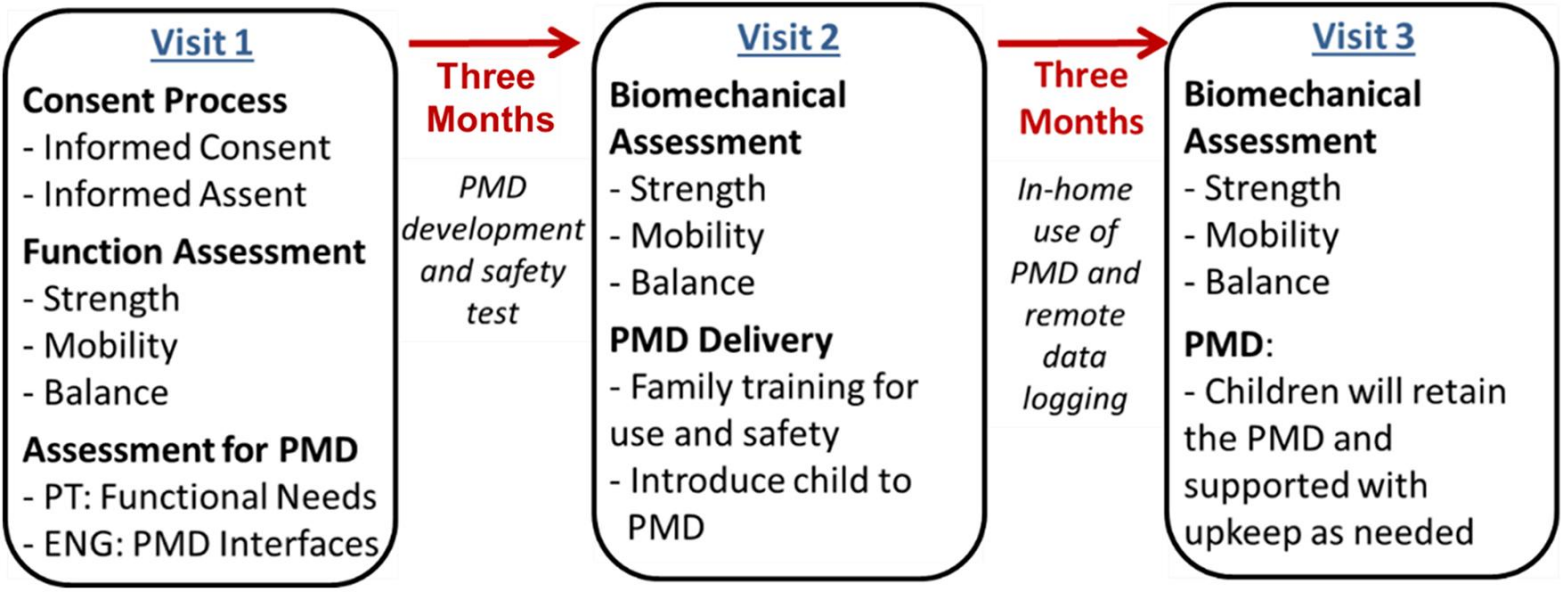
Schedule of participant involvement in research activities.

The intervention period will be three months in which children will take the individually adapted PMD home for use. Figure 1 displays the schedule of enrollment, waitlist period, intervention, and assessments regarding our research activities {13}. Usage is encouraged at home, neighborhood, parks, school, and any other places where the family usually attend. Actual time of device use will be tracked with embedded sensors and stored using local hardware memory devices within the ride-on without any identifier information. No GPS sensors or video/photo/ audio recording devices will be used in any of the mobility devices. The sensor data will be collected during visits by trained CITI-certified study personnel from the home or other location where PMDs may be kept/used. These data will be complemented by parent-report via written log. Bi-weekly follow-up phone calls will be given to maintain rapport with all families.

### Assessment procedures and outcome measures

The primary aim of this study is to determine whether a full-scale RCT is feasible. The feasibility of a future RCT will be determined using the following progression criteria:

- Recruitment Feasibility: At least 70% of eligible participants consent to enroll within the recruitment period.
- Retention Rate: At least 80% of participants complete all study visits and assessments.
- Adherence: Participants use the stand-on PMD for an average of at least 20 min per day based on device tracking.
- Variability of Primary Outcomes: Standard deviation of biomechanical outcomes should allow for a sample size estimation in a full-scale trial.

#### Secondary (exploratory) outcomes: biomechanical variables

While static balance (37–40) contributes to standing/seating stability and function, the dynamic and reactive components (38–41) are crucial for children to engage in life activities such as navigating through busy school hallways. Secondary, exploratory biomechanical outcome measures organized by assessment task are outlined in Table 1.

**Table 1:** Detail of secondary, biomechanical outcome measures.

Approach	Variable	Level of outcome
Static Balance: NIH Toolbox Standing Balance Test		
Kinetics	• COP sway area (cm^2^) and velocity (m/s) • RMS of COP sway (cm)	• Primary • Primary
Electromyography	• Thigh co-contraction* (Quads:Biceps Fem) • Leg co-contraction (Gastroc:Tibialis Ant) • Peak activity (%movement cycle) of distal vs. proximal muscles	• Secondary • Secondary • Secondary
Dynamic Balance: Five-Times-Sit-to-Stand Test and Modified Timed Up and Go		
Kinematics	• Trunk-pelvis coupling (°) during turning step of mTUG	• Secondary
Kinetics	• Time (ms) to reach peak vGRF (5TSTS) • COP sway area (cm^2^) (5TSTS, mTUG turning step) and velocity (m/s) (5TSTS) • RMS of COP sway (cm) (5TSTS, mTUG turning step)	• Secondary • Primary (5TSTS) • Primary (5TSTS)
Electromyography	• Thigh co-contraction* (Quads:Biceps Fem) • Leg co-contraction (Gastroc:Tibialis Ant)	• Secondary • Secondary
Gait: 10-Meter Walk Test, Self-Selected Speed and Fast Pace		
Kinematics	• Hip posture (°) at terminal stance • Trunk posture (°) at initial contact	• Secondary • Secondary
Electromyography	Peak, mean, and duration (%Gait Cycle) of: • Gastrocnemius, Tibialis anterior • Biceps femoris, Vastus Lateralis • Gluteus Maximus	• Secondary • Secondary • Secondary
Spatiotemporal Characteristics	• Speed (m/s) • Single limb stance time (%Gait Cycle)	• Secondary • Secondary

5TSTS, five-times-sit-to-stand test; COP, center of pressure; Gastroc:Tibialis Ant, ratio of gastrocnemius to tibialis anterior; mTUG, modified timed up and go; Quads:Biceps Fem, ratio of quadriceps femoris to biceps femoris; RMS, root mean square; vGRF, vertical ground reaction force.

* Unitless.

### Instrumentation

All biomechanical assessments will be conducted in the Laboratory of Applied Biomechanics and Engineering at the University of North Florida. Three-dimensional kinematic data will be collected using a 12-camera Vicon motion capture system (Vicon Motion Systems Ltd., Oxford, UK) consisting of 12 Vero cameras and 6 FLIR cameras, sampling at 120 Hz. Kinetic data will be collected using four Bertec force plates (464 × 508 mm; Bertec Corporation, Columbus, OH) embedded in the center of the lab's 78 m^2^, sampling at 1,000 Hz. Muscle activity will be recorded using a 16-channel wireless surface electromyography (sEMG) system with Trigno Avanti sensors (Delsys Inc., Natick, MA), sampling at 2000Hz. All three data acquisition systems will be synchronized using Vicon Nexus 2.0 software (Vicon Motion Systems Ltd., Oxford, UK). Spatiotemporal gait characteristics will be captured using a 4’ × 26’ Zeno Walkway pressure-sensing mat (ProtoKinetics LLC, Havertown, PA) with the associated data processing software ProtoKinetics Movement Analysis Software (PKMAS).

### Assessment protocols

Biomechanical assessments will be performed at three timepoints (Figure 1): baseline (3 months prior to intervention), pre-intervention (same day as child receives the individually adapted PMD), and post-intervention (3 months after receiving the individually adapted PMD). Reflective markers will be placed on anatomical landmarks according to a modified Plug-in-Gait model for full-body kinematic tracking. Surface EMG electrodes will be placed bilaterally on tibialis anterior, medial gastrocnemius, rectus femoris, biceps femoris, and gluteus maximus, following SENIAM guidelines.

The following standardized protocols will be administered:

***Static Balance***: NIH Toolbox Standing Balance Test (42). Participants will stand barefoot on a single force platform in a standardized stance (feet hip-width apart, arms at sides) for up to 50 s with eyes open, followed by 50 s with eyes closed. Three trials will be collected for each condition, with rest periods between trials as needed. These two conditions will also be performed with children standing on an unstable surface (Airex®). Tandem stance will also be attempted per protocol.

***Dynamic Balance***: Five-Times-Sit-to-Stand Test (5TSTS) (43) and Modified Timed Up and Go (mTUG) (44). For the FTSTS, participants will be seated on a height-adjustable bench (allowing for 90° of hip and knee flexion) with feet resting on the force plate. Participants will be instructed to stand up and sit down five times as quickly as possible with arms crossed over the chest. Three trials will be collected with rest periods as needed. Hand support will be provided for those who cannot independently rise from the bench. For the mTUG, participants will rise from the seated position, walk 3 meters at a self-selected pace, turn 180°, return to the chair, and sit down. The turning period of the task will be captured on the force plate. Three trials will be collected with adequate rest between trials. Hand-held support will also be provided for those who are unable to walk independently.

***Gait***: 10-Meter Walk Test (45). Participants will walk along the instrumented walkway at self-selected comfortable speed and at fast pace. Three trials will be collected for each speed condition, with rest periods as needed. Hand-held support will be provided for those who are unable to walk independently.

### Relevant concomitant care permitted or prohibited during the trial

Given the period of participation of six months, children will be allowed to receive any standard care deemed necessary by their respective physicians without prohibition while taking part in the study. If a major, surgical intervention is to be scheduled, e.g., selective dorsal rhizotomy, we will discuss options with the parents whether the major intervention can be performed once the study is completed, or whether the intervention must be received prior to participation. Other procedures related to standard care are not prohibited.

### Data processing

Kinematic and kinetic data will be processed using Vicon Nexus 2.0 software for marker labeling, gap filling, and initial data quality checks. Subsequently, biomechanical calculations including hip, knee, and ankle joint angles, as well as trunk and pelvis segment angles, kinetic variables, and lower extremity muscle activity patterns will be performed using custom scripts developed in Python (version 3.11) and MATLAB (R2025a, MathWorks, Natick, MA). Whole-body coordination analysis, including trunk-pelvis coupling using vector coding methods, will be performed using *vailá*, an open-source multimodal motion capture toolbox (46). Raw sEMG signals will be band-pass filtered (fourth-order Butterworth), full-wave rectified, and smoothed using a root-mean-square algorithm with a 50-ms moving window. EMG amplitude will be normalized to peak activity achieved during static standing for each muscle. Co-contraction indices will be calculated as the ratio of flexor to extensor muscles during specified movement phases (e.g., rising from the seat during FTSTS).

#### Biomechanical variable definitions

The following operational definitions apply to the biomechanical variables presented in Table 1:

- Center of pressure (COP) sway area (cm^2^): Total area encompassed by the COP trajectory during the assessment period, calculated using a 95% confidence ellipse method.
- COP velocity (cm/s): Mean velocity of COP displacement, calculated as total COP path length divided by trial duration.
- Root mean square (RMS) of COP sway (cm): A measure of COP variability representing the average deviation of the COP from its mean position throughout the trial.
- Trunk-pelvis coupling angle (°): The relative phase angle between trunk and pelvis segments during turning movements, calculated using vector coding analysis to quantify intersegmental coordination timing and magnitude.
- Co-contraction index (unitless): The ratio of antagonist to agonist muscle activation during specified movement phases, with higher values indicating greater simultaneous activation and potentially less efficient motor control.
- Time to peak vertical ground reaction force (ms): Duration from movement onset (defined as the first rise in vertical force exceeding 5% of body weight) to the maximum vertical ground reaction force during the sit-to-stand transition.

### Statistical treatment

Due to the pilot nature of this study, descriptive statistics will be used to summarize feasibility outcomes, including recruitment, adherence, and retention rates. Variability in biomechanical measures will be estimated using means, standard deviations, and confidence intervals to guide sample size calculations for a future RCT. Given the small sample size, formal hypothesis testing will not be conducted. Preliminary pre-post comparisons of biomechanical outcomes will be reported using effect sizes (e.g., Cohen's d) and confidence intervals. Statistical analyzes will be performed with IBM SPSS Statistics version 28.0 (IBM Co., Armonk, NY, USA).

An external biostatistician will be contracted to perform all statistical treatments and control for any potential observer bias. The biostatistician will also manage the dataset to adjust for any missing data. We envision the use of multiple imputation where necessary, with assumption that the mechanism of missing data points was missed at random.

### Plans to give access to the full protocol, participant-level data, and statistical code

Public access to the full protocol as well as any developed statistical code will be available upon reasonable request to the corresponding author (PI). Moreover, de-identified participant-level dataset will be deposited and made available to the public through NICHD Data and Specimen Hub (DASH), which has established policies and procedures fully consistent with the NIH Data Sharing Policies and applicable laws and regulations. The final dataset will include the biomechanical outcome variables, demographic, and clinical data associated with each participant. Submitted data will be verified with relevant data and terminology standards as well as policies at NIH, NICHD, DASH, and Cerebral Palsy Common Data Elements (47).

## Discussion

This feasibility study will provide critical information on the practicality of conducting a full-scale RCT on the use of stand-on PMDs in children with cerebral palsy. The findings will inform the design of a subsequent, adequately powered clinical trial that can formally evaluate the intervention's effectiveness. While we anticipate improvements in biomechanical measures following PMD use, the findings should be interpreted as exploratory and hypothesis-generating rather than confirmatory.

While general searches for motorized “mobility toys” or “ride-on cars” yield a high volume of results in the public domain, the peer-reviewed clinical literature remains primarily focused on modified seated devices aimed at enhancing social skills and basic mobility participation (12–14). Our protocol distinguishes itself from these more common seated methodologies by utilizing a stand-on configuration specifically designed for the therapeutic needs of children at GMFCS Level III. Although preliminary work has explored the feasibility of standing in modified toys (10, 17), this study is unique in its focus on the biomechanical factors and quantitative postural stability changes that occur when the stand-on PMD is used as a targeted intervention in natural environments. By shifting from a passive seated posture to an active standing requirement, this protocol addresses a critical gap between recreational play and physiological rehabilitation.

Our proposed feasibility research is unique in that it looks at a quantitative measure of postural stability as children participate in independent self-directed functional activities with stand-on PMD within their natural environment. Additionally, the novel stand-on component derived from the built-in harness is unique and will allow for additional lower extremity loading. As children stand on the stationary surface (i.e., inside the PMD) that is self-directed through space (i.e., translation of the base of support), children must modulate lower extremity strength and proprioception to counteract external forces/perturbations and maintain balance. Preliminary evidence from a case study supports this expectation, showing significant improvement in dynamic balance control following 3 months of stand-on PMD use in a young child with spastic diplegic cerebral palsy (48). Thus, it is expected that our exploratory findings will lead to the future clinical trial testing the hypothesis that regular use of adapted PMD while standing and encountering natural environmental perturbations provides the practice and motor control learning needed to improve postural stability in children with impaired balance and mobility.

Considering the target population of young children with cerebral palsy (GMFCS level III), certain approaches for kinematic evaluations may not be feasible since many may exhibit disorders with processing of sensory information (49). This disorder may impact the placement of kinematic markers on the child's skin, which is a requirement to ensure exact quantification of joint amotions for traditional motion capture systems. Given this potential obstacle towards collecting data to address some of our secondary outcome variables, a multimodal toolbox (46) was developed to facilitate kinematic data collection and further calculations of whole-body coordination (e.g., trunk-pelvis vector coding). This open-source, Python-based platform enables motion capture and movement analysis by integrating signals from diverse sources, including retroreflective motion capture technologies, markerless video capture, inertial measurement units, among other sources, to enhance available biomechanical evaluations for pediatric populations with disabilities.
